# Differentiation between Good and Low-Responders to Intravitreal Ranibizumab for Macular Edema Secondary to Retinal Vein Occlusion

**DOI:** 10.1155/2016/9875741

**Published:** 2016-12-01

**Authors:** Marcel N. Menke, Andreas Ebneter, Martin S. Zinkernagel, Sebastian Wolf

**Affiliations:** ^1^Department of Ophthalmology, Cantonal Hospital Aarau, Aarau, Switzerland; ^2^Department of Ophthalmology, Inselspital, University Hospital of Bern and University of Bern, Bern, Switzerland

## Abstract

*Background*. Ranibizumab is approved for treatment of macular edema in eyes with retinal vein occlusion (RVO). Some eyes show low-response to treatment with regard to visual acuity gain (VA) and OCT central retinal thickness (CRT) reduction. The goal of this study was to quantify the percentage of low-responders.* Methods*. Treatment of naïve eyes with macular edema secondary to RVO was included and monthly VA and CRT were analyzed. Four weeks after the loading phase, and at the end of the study, eyes were grouped into low- and good responders based on predefined criteria. The responder and low-responder groups were then compared at various time points.* Results*. Forty-three eyes were included. Regarding VA, 27.9% were low-responders after the loading phase and 30.2% at the end of the study. For CRT, 34.9% were low-responders after the loading phase versus 27.9% at the end of the study. 75% of patients that were VA low-responders and 73.3% of CRT low-responders after loading phase remained low-responders at the end of the study.* Conclusion*. Approximately 30% of patients showed low response to ranibizumab after the loading phase and after 1 year of treatment. Two-thirds of patients that were low-responders after the loading phase remained low-responders after 1 year.

## 1. Background

Central retinal vein occlusion (CRVO) is characterized by blockage of the major outflow vessel of the retina, resulting in retinal hemorrhages, exudates, and macular edema [1]. In branch retinal vein occlusion (BRVO), one of the primary, secondary, or tertiary branches of the central retinal vein are blocked [2]. BRVO leads to similar retinal problems and visual loss due to macular edema if the posterior pole is affected.

Intravitreal injection of ranibizumab (Lucentis; Genentech, Inc., South San Francisco, CA), a Fab fragment that binds all isoforms of VEGF-A, has been shown to markedly reduce macular edema. Two large, multicenter, double-masked trials—the Ranibizumab for the Treatment of Macular Edema following Branch Retinal Vein Occlusion Study (BRAVO) [3] and the Ranibizumab for the Treatment of Macular Edema after Central Retinal Vein Occlusion Study (CRUISE) [4] analyzed the efficacy and safety of intravitreal ranibizumab in either types of retinal vein occlusion. The study results showed that intraocular injections of ranibizumab were beneficial for these patients.

The HORIZON trial (ClinicalTrials.gov identifier: NCT00379795) was designed to obtain additional information about the long-term efficacy of ranibizumab treatment in two patient cohorts. Cohort 1 included patients with neovascular age-related macular degeneration and will not be discussed further. Cohort 2 included patients with macular edema after retinal vein occlusion that had completed the BRAVO or CRUISE studies [5]. Recently, Bhisitkul et al. reanalyzed data from BRAVO and CRUISE to see if optical coherence tomography (OCT) data at baseline or month 3 in the treatment of macular edema had predictive value to differentiate between good and low-responders [6]. They found that at month 3 of ranibizumab treatment, OCT images provided predictive information for patients with CRVO, but not for those with BRVO. Visual outcomes at months 6 and 12 were reduced in patients with CRVO with persistent macular edema at month 3.

The purpose of this study was to analyze data of “real-life” patients outside a clinical trial setting to identify and differentiate low-responders to ranibizumab treatment in eyes with macular edema due to retinal vein occlusion.

## 2. Methods

For this observational, retrospective study, all charts of patients treated at the Department of Ophthalmology, Inselspital, University Hospital Bern, Switzerland, between 01/2010 and 09/2013 with macular edema secondary to BRVO or CRVO have been reviewed to identify eligible patients. Inclusion criteria were macular edema affecting the fovea due to BRVO or CRVO that was treated with repeated injections of ranibizumab. No other prior therapy for macular edema was allowed because eyes that required a switch were already considered as potential low-responder to the previous treatment. All eyes received 3 consecutive injections in the first 3 months of treatment as a loading dose. Monthly follow-up was done over a period of at least 12 months to avoid under-treatment due to delayed examinations. After the loading phase, patients received PRN treatment in case of edema recurrence or new vision loss (>5 letters). At baseline and each following month best-corrected ETDRS visual acuity (VA) and OCT central retinal thickness (CRT, based on the central area of the ETDRS grid) were measured. Final VA and CRT were noted at month 12 or in case of an injection, 4 weeks after the last injection. For OCT retinal thickness measurements, Spectralis OCT volume scans (Heidelberg Engineering, Heidelberg, Germany) were used. The follow-up function was used for repeated measurements. Patients were categorized into a treatment responder and a low-responder group based on VA and CRT change. The first cut-off was made at month 4, four weeks after the loading phase of three consecutive, monthly ranibizumab injections. The second cut-off was made at the end of the observation period. Responder and low-responder groups were then compared. Eyes were characterized as low-responders if VA did not increase >6 letters from baseline, which represents less than 50% of the expected VA gain extrapolated from the BRAVO and CRUISE studies [3–5]. Eyes were also characterized as low-responders if CRT did not decrease >30% from baseline. VA low-responder and OCT low-responder were analyzed separately.

GraphPad Prism software (version 5.02) was used for statistical analysis. Group means of low-responders and responders in VA and CRT at different time points were tested with Student's *t*-test. Differences between good responders and low-responders were tested with the Chi-Square test. *p* values <0.05 were considered statistically significant. All values are presented as mean ± SD.

The study followed the tenants of the Declaration of Helsinki and was approved by the Cantonal Ethics Committee Berne.

## 3. Results

176 charts of patients with macular edema due to CRVO or BRVO were identified. 43 eyes were included in the analysis (22 cases of BRVO; 21 cases of CRVO; 19 females; mean age 71.74 years ± 13.1). All other eyes have been excluded because either they were not treatment naïve or treatment was switched to other intravitreal therapy during the first year. Mean baseline VA was 47.4 ± 17.3 letters. Mean baseline CRT was 588.7 ± 193.9 *μ*m. Final VA increased to 64.0 ± 17.2 letters and CRT decreased to 328.8 ± 139.3 *μ*m. Differences in VA and CRT change were significant (*p* < 0.0001).  Table 1 shows the distribution of patients in the responder and low-responder groups at month 4 and at the end of study. Patients that were low-responders at month 4 as well as at the end of study were counted as a match. The same calculation was performed for responder groups. Results are also shown in Table 1.

**Figure 1:**
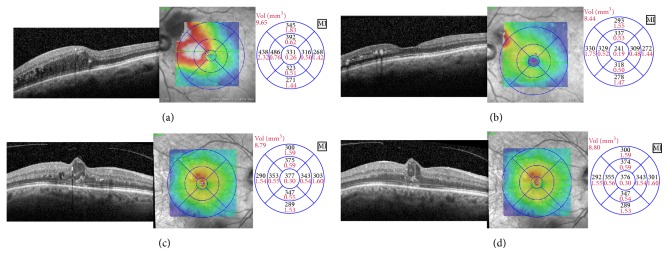
(a) Branch retinal vein occlusion prior to therapy. Macular edema is present in the upper half of the macula. Corresponding color-coded thickness map (middle) and ETDRS mean retinal thickness values are shown (left). (b) 4 weeks after the loading phase (3 monthly injections of ranibizumab) the edema disappeared. Intraretinal exudates are still present. Central retinal thickness decreased from 331 to 241 *μ*m. ETDRS visual acuity improved from 42 to 75 letters. (c) OCT findings in central retinal vein occlusion prior to therapy. A larger cyst can be appreciated in the foveal area. Central retinal thickness is 377 *μ*m. (d) After the loading phase, central retinal thickness remained virtually unchanged. The cystic appearance of the retina is still present. ETDRS visual acuity improved from 45 to 48 letters.

**Figure 2:**
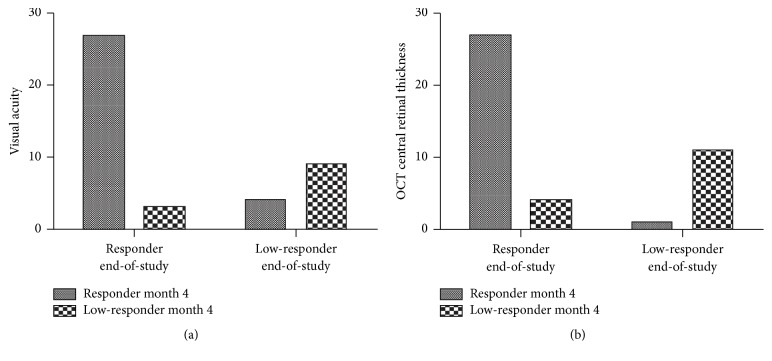
Distribution of responder and low-responder at different time points during the study in regard to visual acuity (a) and OCT central retinal thickness (b). The *y*-axis represents the actual number of patients in each group.

Figure 1 shows typical OCT scans at baseline and month 4 of one patient in the responder and another patient in the low-responder group. Chi-square test revealed significant differences within responder and low-responder groups at month 4 and at the end of study for VA (*p* = 0.0002) and CRT (*p* < 0.0001). The corresponding bar plots are shown in Figure 2.  Table 2 shows mean VA and CRT values at baseline, after the loading phase and at the end of study as well as other characteristics for responder and low-responder patients.

### 3.1. Subgroup Analysis Regarding VA Low-Responder

Group comparison revealed that VA low-responders after the loading phase required significantly more injections within the first year of treatment (10 ± 2 versus 8.4 ± 2 injections; *p* = 0.0067). VA low-responders after the loading phase started with a significantly better baseline VA (58.5 ± 14.8 versus 43.1 ± 16.4 letters; *p* = 0.027) compared to treatment responders. Final visual acuity was significantly lower in the VA low-responder group at the end of study (51.2 ± 15.0 versus 69.4 ± 15.6 letters; *p* = 0.0173). In addition, low-responders at the end of study already showed significantly lower VA values after the loading phase (53.1 ± 16.1 versus 67.2 ± 13.5 letters; *p* = 0.0061). Further, VA low-responders after the loading phase had significantly lower VA values compared to VA responders after the loading phase (56.5 ± 18.2 versus 65.6 ± 13.6 letters; *p* = 0.031). At the end of study, VA low-responders after the loading phase and at the end of study showed significantly higher CRT values at the end of study (final CRT for VA low-responder loading phase: 431 ± 194.5 *μ*m versus 289.2 ± 86.7 *μ*m; *p* = 0.0216, and final CRT for VA low-responder end of study: 417.7 ± 205.1 *μ*m versus 297.6 ± 85.5 *μ*m; *p* = 0.0443).

Finally, VA low-responders after the loading phase and at the end of study showed significantly higher CRT values after the loading phase (474.2 ± 242.3 *μ*m versus 281.0 ± 77.0 *μ*m; *p* = 0.0003; and 464.7 ± 249.3 *μ*m versus 287.7 ± 77.4 *μ*m; *p* = 0.001, resp.).

The distribution of BRVO and CRVO cases in the VA low-responder group was equal (50%). Two low-responder patients showed ischemic areas with capillary dropout in the macula (16%; 1 BRVO, 1 CRVO) during fluorescein angiography, versus 4 patients in the responder group (13%, 3 CRVO, 1 BRVO).

### 3.2. Subgroup Analysis Regarding CRT Low-Responder

CRT low-responder received a mean number of 9.2 injections versus 8.8 injections in the nonresponders group. In addition, CRT low-responders showed significantly higher CRT values compared to responders after the loading phase (458.1 ± 226.9 *μ*m versus 268.9 ± 53.1 *μ*m; *p* = 0.0001) and at the end of study (471.9 ± 196.0 *μ*m versus 288.9 ± 87.9 *μ*m; *p* = 0.05). If patients were CRT low-responders already after the loading phase, mean CRT values were also significantly higher at the end of study (420.9 ± 279.4 *μ*m versus 279.4 ± 68.6 *μ*m; *p* = 0.0083). In addition, CRT low-responders after the loading phase showed significantly lower VA values after the loading phase (57.6 ± 14.3 versus 66.0 ± 15.3 letters; *p* = 0.0001). CRT low-responders at the end of study already showed significantly lower VA values after the loading phase (56.6 ± 13.1 versus 64.9 ± 14.7 letters; *p* = 0.013).

In the CRT low-responder group, 55% of cases had CRVO and 45% BRVO. Two low-responder patients showed macular ischemia (18%; 1 BRVO, 1 CRVO), versus 4 patients in the responder group (12%, 3 CRVO, 1 BRVO).

No other significant differences between the low-responder and responder groups were found for age, baseline VA, or baseline CRT.

## 4. Discussion

Low-response to antiVEGF treatment is frequently observed in clinical routine. Just recently, Guber et al. described certain clinical risk factors for low response to ranibizumab in exsudative age-related macular degeneration [7]. For macular edema secondary to retinal vein occlusion, it remains challenging to identify low-responders early during the treatment process to avoid ineffective and expensive treatment without significant benefit for the patient. Our analysis revealed that, outside of the clinical setting of BRAVO and CRUISE studies, approximately one-third of patients were classified as low-responders for VA gain and CRT reduction after the loading phase and after 1 year of treatment based on our low-responder criteria. Approximately two-thirds of patients that were low-responders for VA and CRT after the loading phase and remained low-responders after 1 year of treatment. These numbers appear to be in the same range as shown by Bhisitkul et al. analyzing low response to ranibizumab treatment in the BRAVO and CRUISE study population [6]. However, criteria for low-response are not uniformly defined for response or low-response in anti-VEGF treatment for macular edema due to retinal vein occlusions. We defined VA low-response as a VA gain of <6 EDTRS letters, which represents less than 50% of the expected VA gain shown in BRAVO and CRUISE studies. This cut-off is somewhat arbitrary and one can discuss if VA low-response criteria are too harsh or if even a VA gain of, for example, 8 letters should be considered a low-response based on the prospective study data of BRAVO and CRUISE [3, 4]. The same argumentation accounts for reduction in CRT. Less than 30% reduction of CRT was chosen as the definition for low-response. Many eyes that are treatment naïve show significant macular edema with CRT values more than twice as high as unaffected eyes. In our study, no significant differences were found in baseline CRT between responder and low-responder at any time point. However, there was a tendency of thinner baseline CRT values in the low-responder group. One can argue that these patients are less likely to show a reduction of CRT of >30% since they start with thinner CRT. The same accounts for VA gain. Patients starting with higher baseline VA values are less likely gaining letters than patient with very low baseline VA values. In fact, when looking at VA low-responders and responders after the loading phase, low-responder patients had a significant higher VA at baseline (58.5 versus 43.2 letters; *p* = 0.027).

Nevertheless, a certain percentage of retinal vein occlusion patients show low-response to anti-VEGF treatment. Noma et al. published two studies measuring VEGF and Interleukin 6 (IL6) levels in the vitreous of eyes with CRVO and BRVO [8, 9]. Mean levels of VEGF and IL6 were increased in these eyes and correlated with the extent of retinal nonperfusion and the severity of macular edema. In addition, these studies revealed that approximately 30% of eyes showed similar (normal) VEGF values that were comparable to healthy controls. It might be coincidental that the numbers of eyes with retinal vein occlusion and “normal” VEGF levels in the “Noma studies” match our numbers of anti-VEGF low response. On the other hand, one can hypothesize that anti-VEGF treatment in eyes with normal VEGF values might not be as effective as in eyes with high VEGF levels. To prove this hypothesis, a prospective study would be required which includes VEGF level measurements prior to treatment. In our study population, no VEGF measurements have been performed.

Recently, new drugs became available for treating macular edema in eyes with retinal vein occlusion. Ozurdex™, a biodegradable dexamethasone implant has been shown to be effective in reducing macular edema and improving patient's vision in a prospective randomized trial [10]. Although intravitreal dexamethasone has been shown to reduce intravitreal VEGF level, it mainly shows anti-inflammatory actions by blocking phospholipase A2 and inhibition of ICAM, IL6, and other cytokines [11–13]. In addition, intravitreal steroids increase the integrity of tight junctions [14]. One can hypothesize that eyes with macular edema due to retinal vein occlusion with normal VEGF levels in the vitreous might not respond well to anti-VEGF treatment but might profit from anti-inflammatory treatment by intravitreal steroid injection. Recently, Tservakis et al. published a small case series of 10 eyes with persistent macular edema after anti-VEGF treatments that were switched to intravitreal dexamethasone. Nine out of 10 cases showed improvement in best-corrected VA and reduction of CRT [15]. However, a prospective, randomized trial is required to show if a switch from an anti-VEGF to an anti-inflammatory drug is beneficial in low-responder eyes.

This study has some limitations. First, this was a retrospective study design with a relatively small study population. Second, patients that were switched to other intravitreal treatments during the observational period have been excluded in order to assess solely the effect of ranibizumab on VA and CRT. Therefore, the study population is biased and the rate of low-response to treatment might be underestimated. Third, no VEGF levels were assessed in our study group. In addition, other morphological changes or baseline values such as the presence of retinal hemorrhages, hard exudates, or concomitant diseases have not been analyzed.

## 5. Conclusion

In conclusion, approximately 30% of patients with macular edema due to retinal vein occlusion showed a low response to ranibizumab treatment after the loading phase and after 1 year of treatment. Two-thirds of patients that were low-responders after the loading phase remained low-responders after 1 year of treatment. Based on our data, a switch of therapy to another intravitreal drug should be considered after the loading phase if a low response is observed. A prospective, randomized comparative trial is needed to find out which therapy switch might actually be beneficial for our patients.

## Figures and Tables

**Table 1:** 

VA responder at month 4	31 (72.1%)
VA responder end-of-study	30 (69.8%)
VA responder match	27 (87.1%)
VA low-responder at month 4	12 (27.9%)
VA low-responder at end-of-study	13 (30.2%)
VA low-responder match	9 (75%)
CRT responder at month 4	28 (65.1%)
CRT responder at end-of-study	31 (72.1%)
CRT responder match	27 (87.5%)
CRT low-responder at month 4	15 (34.9%)
CRT low-responder at end-of-study	12 (27.9%)
CTR low-responder match	11 (73.3%)

Distribution of responders and low-responders at month 4 and at the end of study. Results are shown for visual acuity (VA low-responder = ≤6 letters gain) and central retinal thickness (CRT low-responder = ≤30% decrease in thickness). Patients that stayed within one group over the whole study period were counted as a match. All values are given in *n* (%).

**Table 2:** Group comparisons between low responder and responder to ranibizumab treatment.

	Mean age	% CRVO	% BRVO	VA baseline	VA after loading phase	VA end of study	CRT baseline	CRT after loading phase	CRT end of study	Number of injections
Low-responder group VA month 4	69.25 ± 15.1	58	42	58.5 ± 14.8^b^	56.5 ± 18.2^i^	59.6 ± 20.1	576 ± 179.4	474.2 ± 242.3^l^	431 ± 194.5^d^	10 ± 2^a^
Responder group VA month 4	72.7 ± 12.4	48	52	43.1 ± 16.4^b^	65.6 ± 13.6^i^	65.7 ± 15.9	593.7 ± 201.9	281.0 ± 77.0^l^	289.2 ± 86.7^d^	8.4 ± 2^a^
Low-responder group VA end-of study	74.3 ± 12.9	50	50	53.0 ± 14.0	53.1 ± 16.1^h^	51.2 ± 15.0^c^	595.4 ± 172.3	464.7 ± 249.3^m^	417.7 ± 205.1^e^	9.8 ± 2.3
Responder group VA end-of-study	70.6 ± 13.5	50	50	45.0 ± 18.5	67.2 ± 13.5^h^	69.4 ± 15.6^c^	595.2 ± 201.4	287.7 ± 77.4^m^	297.6 ± 85.5^e^	8.4 ± 1.9
Low-responder group CRT month 4	70.5 ± 11.1	47	53	53.1 ± 12.1	57.6 ± 14.3^j^	61.4 ± 17.8	499.5 ± 202.9	458.1 ± 226.9^n^	420.9 ± 187.7^f^	9.3 ± 2.4
Responder group CRT month 4	72.4 ± 14.2	54	46	44.4 ± 19.1	66.0 ± 15.3^j^	65.4 ± 17.1	635.5 ± 174.3	268.9 ± 53.1^n^	279.4 ± 68.6^f^	8.6 ± 1.9
Low-responder group CRT end-of study	75.9 ± 6.5	60	40	49.6 ± 10.3	56.6 ± 16.1^k^	55.9 ± 17.7	469.6 ± 216.5	439.3 ± 251.3	471.9 ± 196.0^g^	9.2 ± 2.4
Responder group CRT end-of-study	71.0 ± 14.6	53	47	45.8 ± 18.9	64.9 ± 14.7^k^	66.1 ± 16.4	630.1 ± 169.9	298.7 ± 106.8	288.9 ± 87.9^g^	8.8 ± 2.0

^a^No injections: LR versus R VA loading phase: 0.0067.

^b^Baseline VA: LR versus R VA loading phase: 0.027.

^c^End VA: LR versus R VA end-of-study: 0.0173.

^d^End CRT: LR versus R VA loading phase: 0.0216.

^e^End CRT: LR versus R VA end-of-study: 0.0443.

^f^End CRT: LR versus R CRT loading phase: 0.0083.

^g^End CRT: LR versus R CRT end of study: 0.05.

^h^End VA: LR versus R VA loading phase: 0.0061.

^i^Loading phase VA: LR versus R VA: 0.031.

^j^Loading phase VA: LR versus R CRT loading phase: 0.0001.

^k^Loading phase VA: LR versus R CRT end of study: 0.013.

^l^Loading phase CRT: LR versus R VA loading phase: 0.0003.

^m^Loading phase CRT: LR versus R VA end of study: 0.001.

^n^Loading phase CRT: LR versus R: 0.0001.

Corresponding *p* values (letter coded): LR = low-responder; R = responder; CRT = central retinal thickness; VA = visual acuity.

Values are given ± standard deviation. All nonlettered values showed no significant differences.
